# The Adoption and Impact of Telemedicine on Health Equity: A Narrative Review From the Jamaican Context

**DOI:** 10.7759/cureus.69650

**Published:** 2024-09-18

**Authors:** Annetta V Wishart, Ranim K Hamouda, Entesar Elsaady, Muhammad Rizwan Aslam, Alekya Perala, Safeera Khan

**Affiliations:** 1 Research, California Institute of Behavioral Neurosciences and Psychology, Fairfield, USA

**Keywords:** healthcare policy, primary care, healthcare equity, covid-19 pandemic, telemedicine

## Abstract

Telemedicine rose to popularity during the coronavirus disease 2019 (COVID-19) pandemic but is yet to be fully developed. Hence, this study explores the current status of telehealth in Jamaica, looking at its benefits, challenges with its implementation, the regulatory landscape, and solutions to using this technology. Due to the limited research on this topic, a majority of the sources utilized were gray literature with qualitative and quantitative studies. This review seeks to transform policy and practice, promoting telemedicine as a feasible solution for improving Jamaica’s healthcare quality and access. By comparing telemedicine in Jamaica to a more developed nation like the United States, the review highlights not only benefits but also major challenges, including healthcare disparities due to the digital divide, less advanced technology, privacy breaches, and significant financing required for telemedicine infrastructure, among other barriers to its integration. The analysis advocates for improvement in various areas, such as cybersecurity measures, advanced training for healthcare professionals, further investments in technological infrastructure, refinement of regulatory frameworks and policies, and incorporation of community-based initiatives. This investigation further highlights the need for additional research to gain insights and a broader perspective.

## Introduction and background

Telemedicine is defined as the use of technology to deliver a wide array of healthcare services remotely, including patient assessment, diagnosis, and management [1]. The coronavirus disease 2019 (COVID-19) pandemic has encouraged and increased telemedicine adoption globally, underscoring its benefits and drawbacks [1]. Jamaica has many advantages, including decreased healthcare delivery time and relieving overburdened healthcare facilities [2]. As the implementation of telehealth seeks to enhance healthcare access, rural and underserved communities in Jamaica have had major benefits [3]. This highlights its potential in promoting equitable care. It has also merged well into the healthcare systems of developed countries like the United States, supported by digitalization and comprehensive policies [4]. Jamaica has been partnering with local and international organizations to explore using telemedicine to improve the healthcare needs of its population. Despite these efforts, many challenges persist, including socioeconomic, infrastructural, and policy barriers, making it difficult to adapt to these new technologies [3].

Given the limited local research on this topic, this narrative review aims to investigate further and increase awareness about the adoption and impact of telemedicine on health equity in Jamaica. It draws on comparing a more technologically advanced nation like the United States to highlight specific challenges and opportunities for improving our healthcare system. By examining the current state of telemedicine in Jamaica, this review also seeks to contribute to the ongoing discourse on telehealth and its role in creating an inclusive healthcare system.

## Review

Methodology

Aim and Objectives

The research seeks to investigate various aspects of telemedicine in Jamaica while being cognizant of the following research questions: What is the current state of telemedicine in Jamaica? How does telemedicine raise equitable healthcare in Jamaica compared to the United States? What policies influence the implementation of the telemedicine framework? What recommendations exist to improve telemedicine in Jamaica?

Research Design

The review utilized a thematic analysis to thoroughly examine telemedicine in Jamaica. It drew on 19 gray literature sources and two journal articles referencing the Jamaican perspective, and five related to the United States context were obtained from PubMed and ResearchGate. These studies utilized a combination of qualitative and quantitative research methodologies.

Search Strategy

Full-text articles published from 2020 to 2024 were explored, and the search strategy was customized to incorporate current and pertinent literature related to telemedicine in Jamaica and the United States. The keywords utilized for the search included: “telemedicine,” “telehealth,” “telemedicine in Jamaica,” “telemedicine in the United States,” “telemedicine and health equity,” “telemedicine disparities,” “telemedicine policies,” and “telemedicine adoption.”

Findings

Transformative Benefits and Implementation Challenges of Telemedicine in Jamaica

The adoption of telemedicine in Jamaica has significantly enhanced healthcare access, particularly in remote and underserved areas, thereby mitigating geographical barriers to healthcare [3,5]. This is important because 57.2 percent of the Jamaicans resided in the urban areas, while 42.8 percent lived in the rural regions, as evidenced by data at the beginning of 2023 [6]. There was a significant uptake in its use during the COVID-19 pandemic, serving as a critical tool for maintaining healthcare services, with similar trends seen in the United States [1,3]. Telemedicine in Jamaica has been beneficial in addressing various healthcare needs, including minor infections, chronic disease management, mental health support, skin conditions, medication management, and preventive care [7]. Patient triage, preoperative and postoperative care, and remote patient monitoring are additional benefits it offers [8]. With electronic medical records (EHR), telehealth has facilitated the continuity of care by sharing necessary health information in a hospital setting where patients see various specialists, improving care [9]. It has reduced waiting times, facilitated consultations outside regular hours, and eased strained hospital resources [2,3]. This also includes compensating for shortages in the number of healthcare workers [3,9]. On the other hand, the United States has seen a reduction in unnecessary hospitalizations and readmission penalties due to telemedicine implementation [4].

In May 2018, the Minister of Health and Wellness launched a telemedicine project in Kitson Town, a community in rural Jamaica. The program was designed to reduce patient care delivery time to underserved residents of rural communities by providing specialized care remotely [2,10]. The Caribbean Training and Education Centre for Health (C-TECH), in partnership with the Ministry of Health and Wellness, engineered training programs for integrating telemedicine in human immunodeficiency virus (HIV) treatment sites. Of note, the United States Department of Health and Human Services Health Resources and Services Administration (HRSA) has provided funding for these projects [11]. Another advantage of telemedicine is the Sickkids-Caribbean Initiative (SCI), which has an established collaboration with specialists at the Hospital for Sick Children (Sickkids) in Canada and healthcare professionals across six Caribbean countries. The University Hospital of the West Indies and Bustamante Hospital for Children in Kingston, Jamaica, are among the seven telemedicine facilities in the region that are part of this initiative. The local healthcare capacity has been boosted by the training of over 40 oncology and hematology professionals under the SCI, which has supported diagnostic screening and research [3,12]. The contribution of digital devices and the provision of the U-Matter chatline from the United Nations International Children's Emergency Fund (UNICEF) has played an integral role in offering mental health support by facilitating flexible and anonymous counseling options [13]. In addition, access to cardiac care in Jamaica has been broadened through integrated technology, which has allowed remote consultations with specialists from other countries, enabling joint partnerships and second opinions [14].

Jamaica is still in the entry phase of telemedicine due to the many challenges, such as socioeconomic and technological factors plaguing the healthcare industry. This includes disparities in accessing technology and high-speed internet, lack of digital literacy, and the preference for traditional face-to-face health visits [3,15]. However, in the United States, only 23 percent of adults still lack access to high-speed internet service, and under-resourced populations can experience increased inequality in care if limited to face-to-face video consultations [16]. Some Jamaicans, particularly the elderly, are usually not digitally literate, making it difficult to navigate the platforms and be proficiently involved [3]. This is a similar finding among elderly Americans; some rely on family members to assist them with engaging these platforms or are forced to reschedule their appointments for an in-person visit [17]. Physical, communication, and accessibility barriers tend to be more prevalent in the disability community, which faces various forms of discrimination and social exclusion in the traditional healthcare system in Jamaica [3]. However, Americans with disabilities are three times less likely to report using the internet, which is seen in ethnic or racial minority groups and individuals with limited English proficiency [17]. This is due to the technical challenges of integrating interpreters; video visits were particularly difficult for patients with language barriers. Patients with visual or auditory impairments also face limitations [17]. In Jamaica, telemedicine is admittedly costly due to the acquisition and maintenance of hardware and software and adequately trained human resources. Limited funding impedes the integration of telemedicine as it contends with other significant healthcare demands and projects [3]. Access to facilities that offer telemedicine services is also complicated because entities that offer telehealth services are often too expensive for people seeking medical care, especially those without health insurance [3]. Both countries are of the view that telemedicine has reduced the financial constraints associated with in-person visits [3,17]. However, some Americans think that access to telemedicine is for those members of the population who can engage the services independently, have health insurance, or are out of pocket and can afford the necessary equipment to engage, especially for video visits. They also think it benefits persons with a higher socioeconomic status or those with easy access to technology [17].

The World Health Organization (WHO) recognized various hindrances to the global adoption of telemedicine, including concerns regarding medico-legal and ethical issues surrounding doctor-patient confidentiality, data privacy, and other legislative complexities [2]. Poor cybersecurity practices, which entail inadequate encryption and insecure media communication have led to data breaches namely unauthorized eavesdropping during consultations. This exposure undermines patient privacy and trust [2]. Other areas of weakness in the healthcare system are augmented by the connection of numerous devices and antiquated software. Unlike the 2021 cyberattack at a well-known hospital in the United States, Jamaica has yet to encounter major data breaches in its healthcare sector. This event has emphasized the immediate demand for a robust cybersecurity system and the reassessment of security measures across the industry [18].

The mandatory request of hard-copy prescriptions for record-keeping by the archaic Pharmacy Act is a major drawback in implementing telehealth services in Jamaica. This Act (Regulation) of 1975 permits verbal or electronic communication of prescriptions but needs validation in writing within 36 hours. This stipulation often results in some patients failing to retrieve the original script [19]. This also causes a delay in obtaining life-saving medication for those who travel between parishes [20]. Regardless of geographical location in the United States, continuous care can be provided for patients, and obtaining professional second opinions is facilitated by the interstate licensure compacts and knowledge of state laws and legislation [16,21]. Also, there has been some apprehension regarding telehealth indemnification as it only covers traditional face-to-face consultations, and there is no existent system for health insurance claims to be completed and submitted online [20].

The Concept of Telemedicine as a Tool for Raising Health Equity

Improving healthcare access and enhancing health equity in Jamaica and the United States are primary examples of how telemedicine is a critical tool globally. It can present distant health facilities, goods, and services to the most vulnerable patients who benefit both physically and economically [3]. Telemedicine has made an alliance between stakeholders across space and time possible thereby raising equity through efficient patient-centered care [1]. In addition to improving clinical care, telehealth has contributed to a broader health system function, entailing patient education and supply chain management [1].

The challenges of developing a resilient and equitable health system are multifaceted. A few of the many hindrances Jamaica and the United States encounter are limited access to technological devices and broadband services, especially in remote and underserved areas, inadequate digital literacy, and cost [3,16]. However, Jamaica still has an evolving regulatory regime [3]. While it is critical to identify barriers to developing an unbiased and solid healthcare system, it is just as important to quickly find solutions to these healthcare disparities to boost access to healthcare services [1]. Despite the increased use of telemedicine during the COVID-19 pandemic, it provided a unique chance to utilize the healthcare system to promote innovation, refine logistics, and ensure a critical framework, striving for a fairer and just healthcare infrastructure [1]. It also highlighted that each citizen must participate in developing and growing their country’s health delivery system [8].

Jamaica’s Telemedicine Regulatory Framework

A legislative framework that regularizes data privacy, licensing, legislative mandates, compensation, and credentialing is necessary for the unfolding of the maximal benefits of telemedicine [3]. The Jamaican Standard Specification for Telemedicine (JS 359: 2022) which was published by the Bureau of Standards, stands as the only regulation in the Caribbean region that guides the practice of telemedicine [3,22]. Though there are significant benefits as it presides over the daily operation of medical practice, there are also limitations. These drawbacks are that conformity to the guidelines is voluntary and it does not extend to telesurgery [3]. Within this policy structure, there is also the Data Protection Act 2020 which provides principles that oversee data management including regulating sensitive information and data breaches should they occur [3,18]. There are certain aspects to this act that are not clearly defined, such as the liability of processors, accountability of data controllers, and the right of public interest groups to initiate representative actions. This could lead to data of vulnerable groups being mishandled without a possible course of action [3].

In contrast, the United States has a more well-developed telehealth regulatory regime, which is governed by both federal and state laws. Under the federal agencies is the Department of Health and Human Services, which has a major role in telehealth policies, including the Health Insurance Portability and Accountability Act (HIPAA). This act focuses on strict data privacy and security measures safeguarding patients’ information across all telemedicine platforms. However, continuous adjustments are being made to these policies, especially as the COVID-19 subsides. Therefore, clinicians must keep abreast of these updated changes to remain compliant with these evolving regulations [16].

The Solution to Disparities in Telemedicine

Resolving the disparities in telehealth services is pivotal to improving healthcare equity in its access and utilization. In Jamaica, there have been major investments made in telecommunication technology to increase access across the country through various field tests and pilot projects involving the Ministry of Health and Wellness [2,11]. However, Jamaicans have always been mindful of new technology. Therefore, it is essential to approach the integration of telehealth through broad public support and resolving issues like broadband coverage [23,24]. Without ensuring that these issues are resolved, the digitization of the hospitals alone will decrease the realization of telemedicine’s utmost potential [23]. Building trust and understanding is foremost for the implementation and acceptance of digital transformation. Therefore, active community involvement is also necessary for this ongoing effort [24].

Telehealth needs to be curated to best fit all populations, including the older population, as it is important to consider these individuals' varying socioeconomic and educational backgrounds [3]. It is crucial to coach patients on successfully operating telemedicine portals. This process requires a structured visit beforehand highlighting workflows and developing their basic digital literacy skills [17]. The telehealth system should also incorporate language interpretation to accommodate diverse users [25]. In the United States, including a virtual assistant is valuable in helping those with literacy challenges and difficulty utilizing technological devices [17]. Visiting coaches also help to interact with patients and educate them, allowing them to become more digitally proficient [25]. Another creative idea is the installation of internet booths in shelters and libraries, creating a quiet space with internet service [25]. In July 2022, the Department of Health and Human Services and the United States Department of Justice issued recommendations based on federal laws regarding the fair treatment of persons suffering from disabilities and limited English proficiency to access telehealth [16].

The Jamaican healthcare sector must institute rigid security measures, maximize the latest technologies, and prioritize staff training [18]. This was exemplified by the Caribbean Training and Education Center for Health (C-TECH), which has been preparing healthcare workers with fundamental skills to employ telemedicine [11]. Despite the advancement of telehealth, many patients in Jamaica prefer traditional in-person consultations [2]. However, merging telemedicine with traditional care would benefit persons unable or unwilling to visit a physical establishment for medical attention [5].

Some countries have outdated infrastructure and hospital bed shortages; therefore, governing bodies should upgrade medical facilities by digitizing conventional hospital services [26]. Also, in the Caribbean, including Jamaica, the role of third parties involved in cases of data breaches and system failures should be clearly defined. Legislators should prioritize creating a consolidated and transparent regulatory regimen highlighting the distinguishing features of telehealth in the region, including Jamaica [3]. There need to be clear rules for jurisdiction, guidelines for liability, layout of standardized licensing requirements, malpractice insurance coverage, and measures for data protection. It is also essential that clinicians are well-trained. This contributes to patient safety, maintaining ethical standards, and expanding telehealth services [3].

Services provided through telehealth should be attainable by all and utilized without bias. The right to health should not be jeopardized due to digital innovations, and the standard of care should be the same as that of receiving in-patient services. This entails cases such as domestic violence and child custody matters in which persons may need further support and guidance outside the home [3]. The International Covenant on Economic, Social, and Cultural Rights (ICESCR), of which Jamaica is a part, reinforces the protection, respect, and fulfillment of human rights [3]. An amendment to the antiquated Pharmacy Act of Jamaica is necessary in the face of the progressive trends in adopting newer technology [19]. In comparison to the United States, the COVID-19 pandemic influenced many changes to the rules and regulations that govern telehealth, leading to its expansion and improving access to patients [16].

Discussion

Since its emergence, telemedicine has garnered the most traction during the height of the COVID-19 pandemic. However, little is known about its adoption and impact on health equity in Jamaica. By analyzing the status quo of telehealth, this study seeks to pique the interest of its readers, stimulating further conversation on digital health and its quest to shape an unbiased healthcare system. This investigation also delves into the advantages, drawbacks, regulatory policies, recommendations, and telemedicine as a thesis for promoting health equity. A correlation between Jamaica and the United States was made, highlighting areas that have made significant strides and others that require improvement.

This research shows the transformative benefits and the major challenges, underscoring the complexities of telemedicine’s influence on health equity. Jamaica's remote and underserved regions have always suffered significant challenges, such as infrastructure inadequacies, including limited internet access, which has hindered the expansion of telemedicine. This is significant as a substantial part of the population is also located in the rural region. However, through various joint efforts locally, such as the Caribbean Training and Education Center for Health (C-TECH) and the Kitson Town project, the government has been able to assist with overcoming this geographical constraint. This has enhanced access to healthcare through the expansion of telehealth, and services were maintained even during the COVID-19 pandemic. The Ministry of Health and Wellness also partners with international organizations to provide training and resources, including specialized care and digital devices that contribute to patients' overall wellness through the SCI and UNICEF. Though the United States and Jamaica share some similarities regarding the use of telehealth, what is unique is that the United States has a more advanced infrastructure supported by a well-developed and dynamic regulatory framework. The hospitals have also profited from lower readmission rates, thus avoiding penalties.

Medico-legal, data privacy, and ethical issues pertaining to medical confidentiality are of utmost concern, which further complicates the telemedicine landscape. The Jamaican Standard Specification for Telemedicine (JS 359:2022) and the Data Protection Act 2020 are the present policies that highlight the country's dedication to standards that govern telehealth. However, these regulations have limitations in their scope and application, such as the JS 359:2022 not covering remote surgeries and invasive procedures. The Data Protection Act remains obscure regarding the accountability of processors and data controllers if patients’ information is mismanaged. This mainly affects vulnerable groups, prohibiting them from holding these organizations liable. Another legislative obstacle to acquiring telemedicine is the antiquated Pharmacy Act, which still mandates hard-copy scripts. This is in contrast to the United States, which has a fully developed regulatory system that entails strict measures under the Health Insurance Portability and Accountability Act (HIPAA) that prioritize data privacy and security. Furthermore, their framework is continuously evolving to keep abreast of any changes within the healthcare system, like the sudden emergence of the COVID-19 pandemic. This disparity underscores the significance of Jamaica's up-to-date refining of its laws to secure patients’ information and successfully tackle emerging challenges.

Despite gaining ground in telehealth, some issues are yet to be addressed in Jamaica. Many deterrents, including cultural, social, and economic factors, have afflicted the healthcare system. Many Jamaicans have always been concerned about introducing new technology; hence, most prefer an in-patient visit. To bridge this gap, it is important to build trust through open communication, public engagement, and community-based initiatives to educate patients about the advantages of telehealth and address their concerns. Some patients, especially the elderly, are not technologically savvy to utilize the various platforms, which is a similar finding in the United States as these patients often rely on relatives to assist them. Though Jamaicans with disabilities have benefited from telemedicine through improvements in physical, communication, and accessibility barriers, those Americans encounter a lack of modified features within the devices for these individuals to operate the digital platform. In Jamaica, setting up the telemedicine system is costly, and patients without insurance also cannot gain access to certain private entities that offer telehealth.

In finding solutions to these challenges, Jamaica will harness the all available rewards of telemedicine by implementing targeted strategies. Investing in technological infrastructure, such as increasing broadband access and improving digital literacy, is vital to accomplishing widespread telemedicine adoption. Furthermore, training patients and healthcare professionals to maneuver the telehealth platforms and strengthen cybersecurity measures contribute to the ease of access and a resilient telehealth system. The continued partnerships between the government and organizations locally and internationally will boost the telehealth industry.

By managing these issues, Jamaica can have an outstanding telemedicine framework that respects the guidelines while meeting each patient's unique needs. This will give rise to a more equitable and efficient healthcare system, creating better healthcare outcomes and satisfaction for health professionals and patients.

Limitations

Despite the extensive nature of this investigative narrative review, several limitations must be acknowledged. A significant challenge was the limited number of journal articles, which are often peer-reviewed, and the over-reliance on gray literature, which speaks to the study's theme. A comparison between Jamaica and the United States could not be made on the issue of reimbursements due to the lack of data from the Jamaican perspective. Hence, future research is needed to explore this gap. The interpretation of the various literature incorporated in this thematic analysis runs the risk of subjectivity and potential bias. Hence, numerous and diverse articles were used to gain a broader perspective, thereby reducing confirmation bias and increasing the validity and reliability of the study.

## Conclusions

The increased efforts by the government and its collaborators to boost telemedicine usage in Jamaica have helped significantly to reduce the gap in providing health services to remote and underserved communities. This technology, which became eminent during the COVID-19 pandemic, has been effective in maintaining the continuity of care. A well-structured regulatory body will be key to leveraging this system and reaping the plethora of benefits that can be explored. As the country’s healthcare system continues to heal from the aftermath of the COVID-19 pandemic. This is an opportune time for Jamaica to institute customized public interventions and develop comprehensive policies to address the barriers that have deterred telemedicine adoption. While telemedicine is well-established internationally, it is still relatively new to Jamaica. Stakeholders must keep working assiduously to devise techniques to publicize the receptivity and usage of telemedicine. The Jamaican populace stands to benefit greatly from telemedicine’s potential to contribute to equal access to healthcare for all. This study further highlights the need for additional research to gain insights and a broader perspective.
